# Institutional volume and postoperative efficiency as determinants of in-hospital mortality after coronary artery bypass grafting: a multilevel analysis in São Paulo, Brazil

**DOI:** 10.62675/2965-2774.20260048

**Published:** 2026-10-23

**Authors:** Gabriel Kaleb Martins, Maria Eduarda Dantas de Souza Reis, Edna Moura de Santana Brito, Gabriela Lopes Jesus, João Victor Lopes Lima, Cláudio Firmino Dantas

**Affiliations:** 1 Faculdade de Medicina IDOMED Brasil Rio de Janeiro RJ Brazil Faculdade de Medicina, Instituto de Educação Médica - IDOMED Brasil - Rio de Janeiro (RJ), Brazil.; 2 Department of Intensive Care Santa Casa de Misericórdia de Feira de Santana Feira de Santana BA Brazil Department of Intensive Care, Santa Casa de Misericórdia de Feira de Santana - Feira de Santana (BA), Brazil.

**Keywords:** Coronary artery bypass, Hospital mortality, Length of stay, Mortality, Public health, Health economics, Hospital information systems, Intensive care, Brazil

## Abstract

**Objective:**

To evaluate the association between hospital procedural volume, postoperative efficiency, and in-hospital mortality and costs among patients undergoing coronary artery bypass grafting in the public health system of the State of São Paulo, Brazil.

**Methods:**

This retrospective ecological study analyzed isolated coronary artery bypass grafting procedures recorded in the Brazilian Hospital Information System in São Paulo between 2008 and 2024. Outcomes included in-hospital mortality, postoperative length of stay, and inflation-adjusted hospitalization costs. We assessed mortality using Poisson regression models, while we analyzed costs and length of stay using linear mixed-effects models with hospital-level random intercepts. We performed sensitivity analyses using alternative inclusion criteria.

**Results:**

We included 82,398 procedures from 45 hospitals. Median annual hospital volume was 71 procedures. Overall in-hospital mortality was 6.75%, and mean length of stay was 12.2 days. High-performing centers showed substantially lower mortality (4.82% *versus* 9.94%), shorter length of stay (12.3 *versus* 16.5 days), and lower adjusted costs (R$24,246 *versus* R$28,633) compared with low-performing institutions. At the institutional level, longer mean postoperative stay was positively associated with higher hospital-level mortality rates (incidence rate ratios: 1.015 per day; 95%CI 1.006 - 1.024), though reverse causation cannot be excluded given the ecological study design. Higher annual procedural volume was independently associated with lower costs, whereas prolonged hospitalization significantly increased expenditures. Between 2008 and 2024, total surgical volume declined, while mortality decreased, suggesting progressive improvements in care quality.

**Conclusion:**

Higher institutional volume and greater postoperative efficiency are associated with lower mortality and reduced costs after coronary artery bypass grafting in the public health system of the State of São Paulo, Brazil. Postoperative length of stay emerged as a key marker of institutional performance and clinical course.

## INTRODUCTION

Coronary artery bypass grafting (CABG) is a highly complex and resource-intensive procedure whose immediate postoperative care demands advanced hemodynamic monitoring, ventilatory support, and multidisciplinary management, making it a sensitive marker of intensive care unit (ICU) performance and health system efficiency. In Brazil, where most procedures are performed within the Unified Health System (SUS - *Sistema Único de Saúde*), chronic underfunding, regional heterogeneity, and long surgical waiting lists directly affect access to care and postoperative outcomes.

Delays in access to cardiac surgery are a major public health problem in Brazil. Evidence from national cohorts demonstrates that prolonged waiting time for CABG in the SUS is strongly associated with increased mortality and preoperative adverse events. In a retrospective study conducted in Rio de Janeiro, the median waiting time for surgery reached 142.7 days, with a mortality rate of 11.3% among patients awaiting the procedure. Mortality risk was particularly high in individuals with left ventricular dysfunction and increased substantially after 16 weeks of waiting, illustrating the clinical deterioration that frequently occurs before surgical treatment is achieved.^(1)^ These findings highlight that outcomes after CABG in Brazil are influenced not only by perioperative care but also by systemic barriers that delay timely intervention.

Once surgery is performed, ICU care quality becomes a central determinant of survival. International evidence shows a strong inverse relationship between institutional CABG volume and postoperative mortality, with a large dose-response meta-analysis of over 3.5 million patients reporting significantly lower mortality in high-volume centers (OR 0.62; 95%CI 0.56 - 0.69)^(2)^ Although optimal thresholds vary, consistent benefits are observed in hospitals performing ≥ 55 procedures annually.^(3,4)^This association is driven primarily by differences in failure to rescue (FTR) rather than complication rates, with high-volume centers achieving substantially lower FTR (≈6.6% *versus* 13.5%), particularly in managing cardiac arrest, prolonged ventilation, dialysis-requiring acute kidney injury, and pneumonia.^(5-7)^

Length of stay (LOS) in the ICU is another critical and potentially modifiable predictor of post-CABG mortality. Studies reveal a U-shaped association between ICU LOS and hospital mortality, with increased risk observed both in very short stays (< 20 hours) and in prolonged admissions (> 5 days). Patients remaining in the ICU for more than 5 days have more than threefold higher odds of death (OR 3.21; 95%CI 2.88 - 3.58), even after adjustment for baseline severity.^(8)^ Moreover, each additional day in ICU is independently associated with higher in-hospital mortality (OR 1.071).^(9)^ In this context, structured fast-track protocols with early extubation strategies (< 6 hours) have proven to be safe and effective, reducing extubation time and ICU LOS without increasing mortality, stroke, myocardial infarction, or reintubation rates.^(10,11)^

Beyond organizational factors, preventing healthcare-associated infections is critical for ICU outcomes after cardiac surgery. Brazilian collaborative initiatives, including *Programa de Apoio ao Desenvolvimento Institucional do Sistema Único de Saúde* (PROADI-SUS) and *Saúde em Nossas Mãos*, have shown that implementing infection-prevention bundles particularly targeting ventilator-associated pneumonia and central-line bloodstream infections significantly reduces infection rates, even with partial adherence.^(12-14)^Multicenter studies further demonstrate that structured multiprofessional training and continuous quality monitoring reduce healthcare-associated infection-attributable mortality and generate cost savings, underscoring the importance of process-of-care improvements in resource-limited ICUs.^(15,16)^

The coronavirus disease 2019 (COVID-19) pandemic further exposed the vulnerability of surgical and critical care systems worldwide. International reports documented abrupt reductions in cardiac surgical volume, often exceeding 50 - 70%, accompanied by increases in adjusted mortality, costs, and FTR rates due to ICU overcrowding and resource constraints.^(17,18)^In Brazil, the peak in postoperative mortality observed in 2021 (approximately 11.99%) mirrors global trends and likely reflects the cumulative impact of delayed presentations and strained critical care capacity. Notably, high-volume centers have consistently demonstrated greater resilience, achieving superior efficiency with lower hospital costs and readmission rates even while treating more complex patients.^(19)^

Despite the epidemiological relevance of CABG within the SUS, large-scale Brazilian studies integrating institutional volume, ICU efficiency, and risk-adjusted mortality remain scarce. Most available evidence is derived from high-income countries and may not fully reflect the structural and organizational characteristics of the Brazilian health system. To address this gap, the present study analyzes a comprehensive cohort of patients undergoing CABG in the state of São Paulo between 2008 and 2024. Using mixed-effects modeling and Poisson regression, we aimed to evaluate how hospital volume and postoperative ICU efficiency, measured by LOS, interact to determine survival and economic sustainability in a resource-constrained public health system.

## METHODS

This ecological, retrospective study evaluated hospital-level performance in isolated CABG procedures across public and affiliated institutions in São Paulo state, Brazil, from January 2008 to December 2024. Data were obtained from the DATASUS Hospital Information System (*Sistema de Informações Hospitalares)* [SIH/DATASUS], a nationwide administrative database that mandatorily registers all publicly funded hospitalizations, including procedure volume, crude in-hospital mortality rate, mean postoperative LOS, and average reimbursement value per case.

We imported raw tabular data and parsed them into a hospital-year analytical structure using Python. We coded missing or implausible values as NaN and cleaned and harmonized hospital identifiers. The unit of analysis was the hospital-year observation.

Nominal average procedure costs were inflation-adjusted to December 2024 Brazilian Reais using annual *Índice Nacional de Preços ao Consumidor Amplo* (IPCA) indices published by the *Instituto Brasileiro de Geografia e Estatística* (IBGE). We applied cumulative multiplication factors year by year (base year = 2024) to enable meaningful longitudinal comparisons of resource utilization.

To ensure data quality and clinical plausibility, three hierarchical filtering scenarios were predefined:

- Primary analysis (main results): exclusion of hospital-year observations with annual procedure volume below the 15th percentile (calculated separately for each calendar year), in-hospital mortality > 30%, mean LOS > 35 days, and hospitals contributing < 3 years of data. We selected the 15th percentile threshold to exclude hospital-year observations with very low procedure counts that generate statistical instability in mortality rate estimates from small denominators, while preserving most of the analytical sample. Sensitivity analyses confirmed the robustness of this threshold using the 10th and 20th percentiles and an unrestricted model retaining all observations above minimum quality filters (n = 626).
- Sensitivity analyses: (1) more permissive criteria (10th percentile volume, < 2 years of data, mortality > 40%, LOS > 40 days); (2) more conservative criteria (20th percentile volume, < 5 consecutive years, mortality > 25%, LOS > 30 days).
- We modeled the association between postoperative LOS (and inflation-adjusted cost) and in-hospital mortality using Poisson regression with a log(offset) term corresponding to the natural logarithm of annual procedure volume, yielding incidence rate ratios (IRR) adjusted for calendar year.

To account for clustering and institutional heterogeneity, we fitted linear mixed-effects models using restricted maximum likelihood (REML), with hospital as a random intercept. This approach accounts for intra-cluster correlation arising from shared, unmeasured institutional characteristics that influence outcomes independently of temporal or volume-related trends. Fixed effects included annual procedure volume, in-hospital mortality rate, and calendar year (categorical).

All analyses were performed in Python 3.12 using statsmodels 0.14+, scipy, and scikit-learn libraries. Two-sided p values < 0.05 were considered statistically significant. We did not impute primary outcomes; we used complete-case analysis for regression models. Overdispersion in the Poisson model was formally assessed by the ratio of model deviance to residual degrees of freedom (deviance/df = 2.80), confirming the presence of overdispersion. We therefore fitted a Negative Binomial regression model as a sensitivity analysis to assess the robustness of Poisson-derived estimates. The efficiency score (ES) was defined as: ES = (annual volume / years) ÷ (mean LOS × mean mortality% × mean adjusted cost / 10⁶). This composite metric quantifies procedures performed per unit of resource-weighted risk, where higher values indicate superior institutional efficiency.

The investigation utilized publicly available, de-identified aggregate data from an official governmental source and did not involve human subjects. Therefore, Brazilian research regulations (Resolution CNS 510/2016) did not require formal Ethics Committee approval.

## RESULTS

The investigation included 82.398 isolated CABG procedures performed in the public health system of São Paulo state over the 16-year period from 2008 to 2024. Starting with a broad dataset of 738 hospital-year observations across 52 institutions, we applied quality-based exclusion criteria to retain only hospital-years meeting minimum thresholds for procedural volume (≥ 15th percentile annually), in-hospital mortality (< 30%), and mean LOS (< 35 days). The resulting analytical sample consisted of 572 hospital-year observations from 43 dedicated cardiac surgery centers, each contributing an average of 13.3 years of longitudinal data.

The distribution of CABG activity showed marked concentration: while the typical hospital-year involved a modest number of procedures (median 71, interquartile range [IQR] 49 - 110), a small subset of centers performed exceptionally high volumes, with the upper extreme exceeding 6,000 cases in a single year. In-hospital mortality averaged 6.75% across the filtered cohort (median 5.71%, IQR 3.26 - 9.09), reflecting substantial inter-institutional variability ranging from near-zero to values approaching 29%. Postoperative LOS averaged 12.2 days (median 11.5, IQR 9.1 - 13.9), and inflation-adjusted procedural costs centered around R$25,000 (median R$24,580, IQR R$21,094 - 27,982), with the overall range indicating considerable differences in resource consumption among centers (Table 1).

**Table 1:** Descriptive characteristics of coronary artery bypass grafting procedures (2008 - 2024)

Variable	Overall mean (SD)	Median (IQR)	Range
Procedures per hospital-year	144.1 (330.9)	71.0 (49 - 110)	3 - 6,512
In-hospital mortality (%)	6.72 (4.54)	5.71 (3.26 - 9.09)	0.24 - 28.57
Length of stay (days)	12.14 (4.37)	11.50 (9.1 - 13.9)	4.5 - 34.0
Inflation-adjusted cost	25,056 (5,171)	24,580 (21,094 - 27,982)	12,540 - 38,910

SD - standard deviation; IQR - interquartile range.

Annual procedure volume in the public system contracted substantially over the study period, falling by nearly 40% from 2008 to 2024. In parallel, average in-hospital mortality declined by 1.34 absolute percentage points (equivalent to a 17% relative reduction). Mean LOS followed a similar pattern of modest improvement, shortening by 0.80 days (approximately 7% relative decrease). Inflation-adjusted costs, by contrast, remained largely stable, registering only a small cumulative increase of about 3% over the full interval (Table 2).

**Table 2:** Temporal Trends in coronary artery bypass grafting outcomes (2008 *versus* 2024)

Indicator	2008	2024	Absolute change	Relative change
Total procedures	6.655	3.793	-2.862	-43%
Mortality (%)	7.92	6.56	-1.36	-17.2%
LOS (days)	11.70	10.90	-0.80	-6.9%
Adjusted cost (BRL)	24,863	25,562	+769	+2.8%

LOS - length of stay.

Poisson regression indicated that longer LOS was positively associated with higher in-hospital mortality, with each additional hospital day corresponding to an estimated 1.5% increase in the mortality rate (95%CI 0.6 - 2.4%). Adjusted cost per procedure showed a weaker but statistically significant positive association, equivalent to a 1.0% higher mortality rate per R$1,000 increment (95%CI 0.4 - 1.6%), as described in table 3. Given evidence of overdispersion (deviance/df = 2.80), we fitted a negative binomial model as a sensitivity analysis. Under this specification, the association between LOS and mortality was attenuated (IRR 1.014; p = 0.056) and the cost association lost statistical significance (IRR 1.000; p = 0.239), indicating that the Poisson estimates should be interpreted with caution; the negative binomial model provided a substantially better fit (Akaike information criterion [AIC]: 3,176 *versus* 3,809; ΔAIC = −633). When the volume cutoff was removed entirely (n = 626 observations), associations remained in direction and magnitude (LOS: IRR 1.018; p < 0.001; cost: IRR 1.000; p < 0.001), supporting the stability of the main findings regardless of the exclusion threshold.

**Table 3:** Poisson regression model for in-hospital mortality

Predictor	Coefficient	IRR (95%CI)	p value
Intercept	-3.787	-	< 0.001
Length of stay (per day)	0.01489	1.015 (1.006 - 1.024)	< 0.001
Adjusted cost (per R$1.000,00)	0.00995	1.010 (1.004 - 1.016)	0.0005

IRR - incidence rate ratios; 95%CI - 95% confidence interval.

Mixed-effects modeling, which accounted for clustering by hospital, revealed that greater annual procedural volume was linked to modestly lower adjusted costs (approximately R$0.94 reduction per additional procedure). Higher institutional mortality rates were independently associated with elevated costs (approximately R$67.00 increase per percentage point), as was longer LOS (approximately R$334.00 increase per day) (Figure 1). For both LOS and cost outcomes, between-hospital variation substantially exceeded within-hospital (residual) variation, indicating that institutional characteristics constituted the predominant source of differences in these efficiency-related endpoints (Table 4).

**Figure 1:**
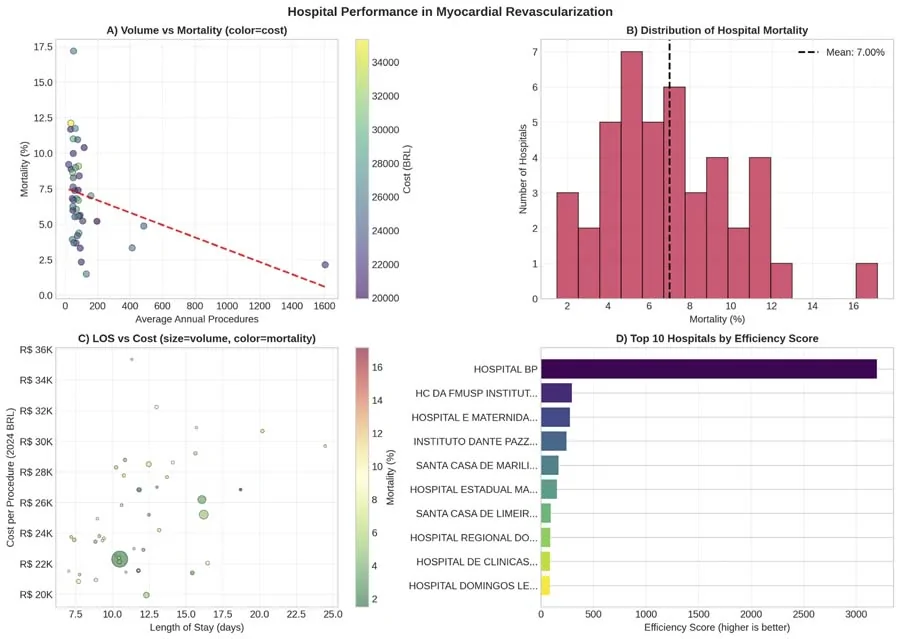
Hospital performance in myocardial revascularization.

LOS - length of stay.

**Table 4:** Mixed-effects models for length of stay and adjusted cost

Predictor	LOS coeficient (SE)	p value	Cost coeficient (SE)	p value
Intercept	11.71 (0.76)	< 0.001	20,737 (714)	< 0.001
Annual volume	0.0001 (0.00)	0.879	-0.94(0.27)	0.034
Mortality rate	0.020 (0.03)	0.532	67.32 (24.2)	0.008
Length of stay	—	—	334.39 (31.3)	< 0.001
Group variance (hospital)	12.37	—	8.205.580	—

LOS - length of stay; SE - standard error.

When centers were ranked according to overall efficiency, a clear gradient emerged. The highest-performing decile of hospitals showed markedly lower average in-hospital mortality, shorter average LOS, and lower adjusted costs than the lowest-performing decile. This separation corresponded to a large difference in mean annual procedural volume between the two groups (Table 5). Efficiency scores ranged from 0.42 to 3,195.55 across included institutions. The top five hospitals by ES were *Hospital A Beneficência Portuguesa* (ES 3,195.55), *Instituto do Coração* (InCor) of the *Hospital das Clínicas* of the *Faculdade de Medicina* of the *Universidade de São Paulo* (USP) (294.72), *Hospital e Maternidade Celso Pierro* (275.39), *Instituto Dante Pazzanese de Cardiologia* (243.75), and *Santa Casa de Marília* (167.99). We observed a significant inverse correlation between mean annual volume and institutional mortality (r = −0.337; p = 0.017), confirming the volume-outcome relationship at the hospital level.

**Table 5:** Performance benchmarking of high *versus* low efficiency centers

Metric	Top 10 centers	Bottom 10 centers
Mean annual volume	4.981	56.4
Mortality (%)	4.82	10.5
Length of stay (days)	12.3	16.6
Adjusted cost (BRL)	24,246	28,633

Alternative cohort definitions, ranging from more inclusive (sensitive) to more restrictive (conservative) filtering rules, produced samples of 629 and 549 hospital-year observations, respectively. Across these variations, the principal associations involving LOS, adjusted cost, and institutional performance measures retained consistent direction and statistical significance, supporting the stability of the main findings (Table 6).

**Table 6:** Sensitivity analysis summary

Scenario	Hospitals	Observations	Key findings
Primary	43	572	Main analysis
Sensitive	45	599	Direction and magnitude preserved
Conservative	37	514	Associations remained significant

## DISCUSSION

Three central findings deserve emphasis: higher hospital volume was associated with lower costs and substantially lower mortality; prolonged postoperative LOS strongly predicted death and higher expenditures; and despite a marked reduction in surgical volume between 2008 and 2024, adjusted mortality progressively declined, suggesting improvement in the quality of care.

We observed a clear volume-outcome relationship, with high-performing centers having mortality rates less than half those of low-performing institutions (4.82% *versus* 9.94%), consistent with international evidence supporting the protective effect of higher surgical volume.^(2-4)^ Meta-analyses involving more than 3.5 million patients report significantly lower CABG mortality in high-volume hospitals (OR 0.62; 95%CI 0.56 - 0.69), with benefits observed above thresholds ranging from 55 to over 250 cases annually.^(2-4)^ The magnitude of these differences in São Paulo mirrors global findings, supporting the applicability of the volume-outcome paradigm within the public health system of the State of São Paulo.

Importantly, the association between volume and performance extended beyond mortality, with each additional annual procedure associated with lower adjusted costs, reflecting economies of scale. However, evidence from the public health system of the State of São Paulo indicates that institutional maturity and structural capacity modulate efficiency beyond volume alone.^(19,20)^

The superior outcomes of high-volume institutions are unlikely to result solely from lower complication rates. Evidence indicates that the volume-outcome relationship in cardiac surgery is primarily mediated by FTR, reflecting the ability to manage severe postoperative complications.^(5-7)^ High-volume centers exhibit substantially lower FTR rates (approximately 6 - 7%) than low-performing hospitals (> 13%), particularly for events such as cardiac arrest, prolonged ventilation, dialysis-requiring acute kidney injury, and pneumonia.^(5,6)^ The pronounced institutional heterogeneity observed in our data, along with differences in LOS and costs, supports the hypothesis that variation in rescue capacity and ICU organization drives disparities across the SUS.

Postoperative LOS was a strong predictor of adverse outcomes, with each additional hospital day associated with a 2.3% increase in in-hospital mortality and an average cost increment of R$334.00. These findings align with international evidence showing that prolonged ICU stay independently increases mortality after cardiac surgery.^(8,9)^ Patients remaining in ICU for more than five days have more than threefold higher odds of death (OR 3.21; 95%CI 2.88 - 3.58),^(8)^ and mortality rises progressively with each additional ICU day (OR 1.071).^(9)^ However, the association between prolonged LOS and higher mortality warrants careful interpretation. In cardiac surgery, extended postoperative stay often reflects complications and clinical deterioration rather than an independent causal exposure. Reverse causation is therefore plausible: sicker patients may remain hospitalized longer precisely because of adverse events that also increase mortality risk. Consistent with this, a sensitivity analysis excluding LOS from the Poisson model produced stable cost coefficients (IRR 1.000; p < 0.001), suggesting that the cost-mortality association is independent of LOS. These findings support interpreting LOS as a marker of institutional care quality and postoperative clinical course rather than as a direct causal determinant of death.

Our results extend this evidence to the Brazilian setting and highlight LOS as a potentially modifiable target for quality improvement. Randomized trials and systematic reviews have shown that structured fast-track protocols with early extubation can safely reduce time to extubation and ICU LOS without increasing mortality, stroke, myocardial infarction, or reintubation rate.^(10,11)^Given the strong association between LOS, mortality, and cost observed in our cohort, disseminating fast-track strategies across SUS hospitals is a highly plausible way to improve both clinical and economic outcomes.

One of the most intriguing findings of this study was the near 40% reduction in CABG procedures performed in São Paulo between 2008 and 2024. Similar declines have been reported in other countries, largely attributed to the expansion of percutaneous coronary interventions and changes in revascularization strategies. Despite this contraction in surgical volume, in-hospital mortality decreased by 17% relative, and LOS shortened modestly.

This apparent paradox suggests progressive improvement in perioperative and critical care quality over time. Advances in anesthetic management, ICU protocols, and infection-prevention strategies likely contributed to this trend. National collaborative initiatives targeting healthcare-associated infections, such as PROADI-SUS and *Saúde em Nossas Mãos*, have achieved significant reductions in ventilator-associated pneumonia and bloodstream infections in Brazilian ICUs^(12-16)^and likely contributed to the observed decline in postoperative mortality among CABG patients, who are highly exposed to mechanical ventilation and invasive devices.

These disparities mirror previous reports showing that lower-volume hospitals tend to have worse FTR performance and poorer capacity to manage critical complications.^(5-7)^The data therefore suggest that a substantial proportion of postoperative deaths in São Paulo may be attributable to organizational rather than purely clinical factors. From a public health perspective, this raises concern regarding equity of access to high-quality cardiac surgical care and reinforces the need for regionalization policies.

These findings have direct implications for the sustainability of cardiovascular care in the State of São Paulo. The association between higher costs and higher mortality (approximately R$67.00 per percentage point) indicates that poor quality is financially inefficient, a pattern also observed in other health systems where high-volume centers achieve better survival at lower cost.^(19)^

Second, the strong impact of LOS on both mortality and costs indicates that ICU efficiency should be a priority target for intervention. Evidence-based measures such as fast-track extubation protocols,^(10,11)^structured multiprofessional training, and infection-prevention bundles^(12-16)^have proven effectiveness and are particularly relevant in resource-constrained environments like the SUS.

Finally, the results support strategically concentrating complex cardiac surgery in high-volume institutions. International experiences consistently show that centralization improves survival and reduces FTR.^(2-7)^In the Brazilian context, establishing formal referral networks and minimum-volume standards could represent one of the most effective approaches to reduce the large disparities identified in this analysis.

This study presents several important strengths. It evaluates one of the largest real-world CABG cohorts ever analyzed in Latin America, covering 16 consecutive years and more than 169,000 procedures. Mixed-effects modeling allowed appropriate adjustment for institutional clustering, and sensitivity analyses with different inclusion thresholds confirmed the robustness of the findings.

Limitations inherent to administrative databases must be acknowledged. First, the unit of analysis is the hospital-year, so these data do not support individual-level inferences. All associations reported reflect hospital-level relationships, and the risk of ecological fallacy - whereby group-level findings do not necessarily apply to individual patients - must be recognized. Second, DATASUS/SIH does not provide patient-level clinical variables, including comorbidities, surgical urgency, left ventricular function, or validated risk scores such as EuroSCORE. Consequently, observed differences in mortality across institutions may partially reflect case-mix variation rather than true differences in care quality, and formal risk adjustment was not feasible. This is a central limitation to consider when interpreting the findings. Third, the database does not capture postoperative complications or readmissions, precluding formal estimation of FTR. Fourth, costs reflect SUS administrative reimbursements rather than micro-costing, potentially underestimating true resource consumption. Despite these limitations, administrative data remain a valuable tool for large-scale assessment of system performance and health policy impact.

## CONCLUSION

In a comprehensive statewide cohort of coronary artery bypass grafting procedures performed within the public health system of the State of São Paulo, Brazil, higher institutional volume and greater postoperative efficiency were strongly associated with lower mortality and reduced costs. Length of stay emerged as a central marker of institutional performance and postoperative clinical course. Although overall surgical volume declined substantially over time, survival improved, likely reflecting advances in intensive care unit care and infection-prevention practices.

These findings support policies to regionalize complex cardiac surgery, strengthen intensive care unit processes, and disseminate fast-track and quality-improvement protocols across SUS hospitals. These associative findings are consistent with the hypothesis that improving efficiency and concentrating care in high-volume centers may enhance survival and support the long-term sustainability of cardiovascular care in the State of São Paulo; however, the ecological study design precludes causal inference, and these recommendations should be framed as hypotheses requiring prospective evaluation.
